# Exploring the Multifaceted Potential of *Elaeagnus angustifolia* L.: A Comprehensive Review of Its Nutritional, Pharmacological, and Environmental Significance

**DOI:** 10.1002/fsn3.70486

**Published:** 2025-07-04

**Authors:** Muhammad Abubakkar Azmat, Aqeel Shahzad, Muhammad Shaban, Asif Ali Khan, Sadaf Shakoor

**Affiliations:** ^1^ Department of Plant Breeding and Genetics University of Agriculture Faisalabad, Burewala Campus Faisalabad Pakistan; ^2^ Plant Breeding Department, INRES University of Bonn Bonn Germany; ^3^ Institute of Plant Breeding and Biotechnology Muhammad Nawaz Sharif University of Agriculture Multan Pakistan; ^4^ Department of Food Science and Technology University of Agriculture Faisalabad, Burewala Campus Faisalabad Pakistan

**Keywords:** botanical description, chemical composition, *Elaeagnus angustifolia*, medicinal importance, nutraceuticals, taxonomy

## Abstract

*Elaeagnus angustifolia* L. is commonly known as oleaster or Russian olive. It is an economically important plant due to its wide applications and ability to thrive under harsh environmental conditions, including various edaphic and topographical circumstances. Different parts of the *E. angustifolia* serve multiple purposes, including food, nutraceuticals, fuel, timber, windbreaks, and erosion control. Furthermore, the abilities of *E. angustifolia* as a nitrogen fixer, bio‐monitoring, and bio‐absorbent characteristics make it an essential component of soil fertility improvement programs. This review elucidates the biochemical characteristics of *E. angustifolia* , encompassing the nutritional and bioactive compounds present in its various parts, including sugars, proteins, minerals, and complex carbohydrates. Additionally, recent findings about the therapeutic potential of *E. angustifolia* , including its analgesic, anti‐inflammatory, antioxidant, cardioprotective, antimicrobial, and anticancer properties, were summarized. Likewise, wound healing effects are described as possible starting points for the development of innovative cosmeceuticals. In short, this review advances current knowledge and provides deep insights into the versatile applications of *E. angustifolia* in focusing on human health concerns and targeted development of nutraceuticals, pharmaceuticals, and environmental sustainability.

## Graphical abstract

*Elaeagnus angustifolia* is a resilient, multi‐purpose plant valued for its roles in food, medicine, fuel, and environmental protection. This review highlights its rich biochemical composition and therapeutic properties, including antioxidant, anti‐inflammatory, and wound‐healing effects. It emphasizes the plant's potential in developing nutraceuticals, pharmaceuticals, and eco‐friendly applications.

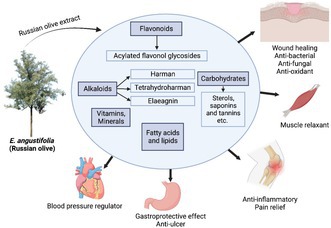

**Abbreviations**
Bcl2: B cell leukemia/lymphoma 2, N‐Histidine‐tagged
DPPH: 2,2′‐diphenyl‐1‐picrylhydrazyl radical
EAE:
*E. angustifolia* water extract

*E. angustifolia*

:
*Elaeagnus angustifolia*

FRAP: fluorescence recovery after photobleaching
HDL: high‐density lipoprotein
HER2: human epidermal growth factor receptor 2
HRE2: human epidermal receptor 2
IL‐6: interleukin‐6
JNK1/2: c‐Jun N‐terminal Kinase 1 and 2
KRAS: Kirsten Rat Sarcoma viral oncogene homolog
LDL: low‐density lipoprotein
LPFI: Lequesne Pain and Function Index
PGA: patient global assessment
SKBR3: Sloan‐Kettering Institute's Breast Cancer 3
SOD: superoxide dismutase
STAT3: signal transducer and activator of transcription 3
TC: total cholesterol
TNF‐α: tumor necrosis factor‐α
VAS: Visual Analog Scale

## 1 Introduction

Medicinal plants and herbs have long been used due to the abundance of valuable bioactive compounds (Mohammadhosseini et al. 2019). Among these plants, *E. angustifolia* (commonly known as Russian Olive) is remarkably known as a beneficial species due to its diverse pharmacological properties. This deciduous, long‐lived, mesophytic, oligotrophic, photophilic, and riparian plant grows near water corridors. It is native to southern Europe and the Middle Eastern and Central Asian regions. This plant has fast growth, and it is well characterized by olive‐like edible nutritious fruit (Fonia et al. 2009). Due to its resemblance to an olive tree, it is also called an oleaster and Russian olive. The leaves, bark, flowers, fruits, and seeds of the *E. angustifolia* plant are being used to prepare decoctions, which are traditionally used to treat different ailments of humans and livestock. Several studies have demonstrated the anti‐inflammatory, antioxidant, cardioprotective, antimicrobial, and anticancer properties of this plant. Also, several phyto‐complexes and juices obtained by *E. angustifolia* have been reported as anticancer, hypocholesterolemic, and cardiovascular protective agents. *E. angustifolia's* flavonoids and phenolic components, which scavenge free radicals, inhibit oxidative enzymes, and chelate metal ions, shield cells from oxidative stress and lower the risk of degenerative diseases (Farzaei et al. 2015, 2019; Niknam et al. 2016; Tehranizadeh et al. 2016; Hamidpour et al. 2017, 2019).

The diverse array of phytochemicals, such as polysaccharides, amino acids, saponins, carotenoids, vitamins, flavonoids, coumarins, phenol carboxylic acids, tannins, and volatile oils in leaves, fruits, and flowers beckons further exploration as a promising reservoir of potential nutritional and nutraceutical compounds (Bucur et al. 2008). This review article comprehensively discusses the taxonomy, distribution, botanical description (e.g., root, leaf, stem, flower, fruit, and seed), phytochemical constituents (for instance carbohydrate, polysaccharides, protein, fatty acids, lipids, phenolic compounds etc.), effectiveness of its extracts for improving sexual health, its multipurpose uses, and recent ethnomedicinal and pharmacological benefits of *E. angustifolia* .

## 2 Taxonomy and Distribution

*E. angustifolia*, with a chromosomal count of 2*n* = 2*x* = 12, is a member of the Elaeagnaceae family. This family comprises three genera: *Elaeagnus*, *Sheperdia*, and *Hippophae*, with a total of 77 species worldwide (Asadiar et al. 2013), primarily found in the Northern Hemisphere's temperate zones, some in eastern Australia and tropical southeast Asia (Bartish and Swenson 2004). *E. angustifolia* is a deciduous and monoecious shrub that grows to a height of 10 m, and its wild form has a denser canopy (Ersoy et al. 2013). The native regions of *E. angustifolia* include the temperate regions of the Mediterranean, southwestern Asia, and Irano‐Turano regions. *E. angustifolia* is supposed to have originated in southern Russia, while in Pakistan and Turkey, it was introduced from Kazakhstan (Gaskin et al. 2019).

*E. angustifolia* is naturally present along the edge of roadsides, fields, mountain slopes, and riverbanks (Azmat et al. 2020). It can be grown under various soil and climatic conditions. Its seedling has the potential to tolerate soil salinity, alkalinity, and acidity (up to pH 6) (Kiseleva and Chindyaeva 2011; Azmat et al. 2020). Its seedlings are also shade tolerant; in contrast, the plant continues to grow in high light intensities. This plant also tolerates different temperature ranges from 45°C to 46°C (Kiseleva and Chindyaeva 2011; Enescu 2018). In recent years, *E. angustifolia* has been promoted in different parts of the world, especially in the USA, Mexico, Canada, and Europe to control erosion, rehabilitate problematic lands, and for ornamental purposes. However, the outstanding regeneration capability of this plant makes it an invasive species in some regions (Katz and Shafroth 2003; Gaskin et al. 2019).

## 3 Botanical Description

The Elaeagnaceae family belongs to the order Rosales of class Magnoliopsida and includes Genus Elaeagnus. This family comprises small trees, shrubs, and flowering plants, which have hairs or thorns on their leaves and have xerophytic and halophytic properties; all genera have nitrogen‐fixing capacity (Nazir et al. 2020). Each genus has a varying numbers of chromosomes: 2*n* = 22, 24, 26, 28, and varies in the reproductive system. Elaeagnus is monoecious, while Hippophae and Shepherdia are dioecious (Bartish et al. 2002).

### 3.1 Roots, Stem, and Leaves

*E. angustifolia* is categorized explicitly by a well‐defined taproot system with highly developed lateral roots. Depending upon the soil type and water availability, the lateral root may reach a depth of 12 m (Zhou et al. 2007). Stubbendieck et al. (2003) have noted that the type of soil, its pH, and even its aeration can greatly influence the level of symbiotic nodulation that takes place on their roots. *E. angustifolia* is predominantly multistemmed and usually possesses 5–6 primary stems that arise from above the crown root surface. The remaining height above ground varies from 4 to 11 m depending on soil and environmental conditions, with approximately 30‐cm trunk diameter at breast height. Branches are randomly arranged and covered with reddish‐brown or silvery hues with coarse thorns (Zhou et al. 2007).

Leaves are petiolate with oblong‐elliptic to lanceolate and occasionally round‐ovate lamina shapes, which are alternatively attached to the plants by twigs. The leaf apex could be acute‐acuminate or round‐obtuse while the leaf margin ranges from revolute to entire, having a round‐obtuse or attenuate‐cuneate leaf base. The presence of silvery scales (speckles) on both the adaxial and abaxial leaf surfaces is one of the characteristic features of *E. angustifolia* leaves. There are variations in leaf shapes both among and between the canopies of individual plants. Leaf length and width range from 1 to 4 cm and 2 to 10 cm, respectively (Klich 2000).

### 3.2 Flower, Fruit, and Seed

Flowering occurs from June to August when it bears silvery‐yellow, attractive, and fragrant campaniform flowers of ~3–12 mm length appearing in the form of small auxiliary clusters. The flowers are entomophilous allogamous and complete with a unilocular superior ovary, apically bent style, sticky stigma and four extensive powdery stamens (Pretty Paint‐Small et al. 2013). Fruits ripen in August or early September forming fleshy, dry‐mealy, and yellow‐red classical drupe with berry‐like oval shape. Fruit length, width and weight range from 10–20 mm, 6–13 mm and 0.5–1.5 g, respectively (Kiseleva and Chindyaeva 2011).

Fruit of *E. angustifolia* contain a single seed, covered with a hard seed coat. The length and width of the seed could be 17 and 4.5 mm, respectively with 0.3–0.35 shape index and 0.2–0.4 g in weight (Katz and Shafroth 2003). Normally, the seeds require two to three months of cold stratification period before germination due to the hard seed coat and the presence of a coumarin‐like inhibiting substance. Viability of seeds is up to 3 years under standard storage conditions. *E. angustifolia* can reproduce either sexually through seed or asexually through root suckers, layering and cuttings (Bonner 2008; Brock 2003).

## 4 Phytochemical Composition

Phytochemical investigations revealed the presence of various bioactive compounds with a range of biological activities; some of these are summarized in Table 1 and are listed below.

**TABLE 1:** Phytochemical composition of different bioactive compounds identified from various parts of *Elaeagnus angustifolia* .

*Elaeagnus* spp. is in the family riparian trees growing near the rivers or water corridors	Percentage in dried ripe fruits of *E. angustifolia*		References
Reducing sugars	50.67%–55.75%		Abizov et al. (2008), Tehranizadeh et al. (2016)
Total sugar	60% ± 5%		
Pectic polysaccharides	3.58% ± 0.3%		
Total flavonoids and polycarboxilic acids	1.35% ± 0.15%		
Total saponins	1.96% ± 0.52%		
Ascorbic acid	5.6 mg%		
B‐carotene	17.5 mg%		
Tannin	5.03% ± 0.05%		
**Composition of the essential oils isolated from the flowers and leaves of *E. angustifolia***	**Flower %**	**Leaves %**	Torbati et al. (2016)
Hydrocarbons	4.69	3.02	
Oxygenated compounds	91.9	95.95	
**Most abundant mineral found in *E. angustifolia* fruit**	**mg/kg**		Hamidpour et al. (2019)
Potassium	8504		
Sodium	1731		
Phosphorus	635		
**Most abundant phenolic compounds found in *E. angustifolia***			
4‐ hydroxybenzoic acid	45.8 mg/100 g dry wt.		
Caffeic acid	32 mg/100 g dry wt.		
Fructose	27.1% dry wt.		
Glucose	22.3% dry wt.		
**Major fatty acids in *E. angustifolia***	**Percentage (%)**		Sahan et al. (2015)
Palmitic acid	34.31		
Oleic acid	26.23%		
Lignoceric	17.47		

### 4.1 Carbohydrates

The characteristic taste of ripened *E. angustifolia* fruit is due to the presence of various carbohydrates. Fructose (27.1%) and glucose (22.3%) are among the major sweetening compounds in *E. angustifolia* fruits, while sucrose is only a minor sugar (Ayaz and Bertoft 2001). Additionally, minor reducing sugars including xylose, mannose, and rhamnose have also been quantified from the extracts of fruit (Abizov et al. 2008).

#### 4.1.1 Polysaccharides

Polysaccharides from fruits, vegetables, mushrooms, and medicinal plants are endowed with many biological activities, including anti‐inflammatory (Wei et al. 2023) antioxidant (Tang et al. 2023) prebiotic (Antunes et al. 2023) antidiabetic (Zhao et al. 2011) and neuroprotective properties (Zhang et al. 2023). Several polysaccharides, including EAP‐1a ( *Elaeagnus angustifolia* Polysaccharide‐1a) and EAP‐1b ( *Elaeagnus angustifolia* Polysaccharide‐1b), were derived from the fruits of *E. angustifolia* . These polysaccharides are mostly composed of mannose, rhamnose, glucose, galactose, xylose, and arabinose. These compounds promote phagocytosis and nitric oxide generation in RAW 264.7 macrophages (Du et al. 2016). It was found that these polysaccharides have antioxidant properties, mainly comprised of xylose, glucose, rhamnose, mannose, and galactose (Chen et al. 2014). Moreover, most polysaccharides present in the fruit of this plant are water‐soluble and possess good emulsifying activity (Sharifian‐Nejad and Shekarchizadeh 2019).

### 4.2 Proteins, Fatty Acids, and Lipids

The fruit of the Russian olive is considered a rich protein source containing both essential and nonessential amino acids (Abizov et al. 2008; Niknam et al. 2016). Their fruit, flower, and seeds are also rich in various fatty acids and derivatives. The flowers of Russian olive primarily contain free fatty acid followed by thioglycolic acid and esters, while the exocarp of the fruit is rich in palmitoleic acid. More than 20 fatty acids of different classes have been quantified from the extracts obtained from *E. angustifolia* fruit pulp (Hamidpour et al. 2017).

To date, several fatty acids (both saturated and unsaturated; glycolipids and phospholipids) have also been found in *E. angustifolia* seeds, with linoleic and palmitic acids identified in seed oil (Ayaz and Bertoft 2001; Hamidpour et al. 2017; Abdalla 2019). Among lipophilic substances, the phytosterols β‐sitosterol and its acetate form were identified in fruits and barks, while stigmasterol was found in the bark (Si et al. 2011). The leaves were also found to contain several lipids, such as fatty acids, triterpenes, sterols, free alcohols, and esters (Bekker and Glushenkova 1997). In the bark, isocaryophyllene was identified, while limonene, nerolidol, squalene, and ursolic acid were extracted from the essential oils of the flowers, and α and β amyrin were isolated from the leaves (Bekker and Glushenkova 1997; Si et al. 2011).

### 4.3 Phenolic Compounds and Flavonoids

Phenolic compounds and their derivatives are widely distributed in different parts of the *E. angustifolia* plant. The concentration of phenolic compounds in the extracts obtained from different plant parts varies with the age and health of the plant and/or time of sampling. Leaves and fruits of *E. angustifolia* represent good sources of phenolic compounds, such as *p*‐hydroxybenzoic, caffeic and protocatechuic acid. Fruits also contain 4‐hydroxybenzoic acid, 4‐hydroxycinnamic acid, benzoic acid, ferulic acid and vanillic acid (Ayaz and Bertoft 2001). Leaves were shown to contain a higher total phenolic content than fruits (Carradori et al. 2020).

Flavonoids are a group of polyphenolic compounds with low molecular weight and high antioxidant activity found in most of vegetables and fruits (Okmen and Turkcan 2014). These secondary metabolites exert many biological effects including antioxidant, anti‐inflammatory, hypocholesterolemic, antidiabetic and neuroprotective properties (Zeka et al. 2017; Ayaz et al. 2019; Farzaei et al. 2019) and may also have a role in cancer prevention (Arroo et al. 2014). *E. angustifolia* has high amounts of flavonoids including flavanol, flavanone, proanthocyanidin, isoflavone and anthocyanin in leaves, fruits and flowers (Hamidpour et al. 2019). The leaves contain flavonoids such as (+)‐gallocatechin, 4 (+)‐catechin, (−)‐epigallocatechin, (−)‐epicatechin, kaempferol, quercetin, luteolin, isorhamnetin‐3‐d‐gluco‐d‐galactoferuloyl, isorhamnetin‐3‐rhamnoglucorhamnoside and isorhamnetin‐3‐d‐gluco‐d‐galactoside (Hamidpour et al. 2017, 2019). In addition to the already known flavonoids, various novel flavonoids such as 4 (+)‐catechin, (−)‐epicatechin, (+)‐gallocatechin, (−)‐epigallocatechin, quercetin, luteolin, isorhamnetin and isorhamnetin‐3‐*O*‐β‐d‐galactopyranoside were isolated from the leaves of *E. angustifolia* (Yuca et al. 2021). These molecules were also detected in fruits of *E. angustifolia* . Particularly, different flavonoids such as rutin, eleagnoside, isorhamnetin (3, 5, 7, 4‐tetrahydroxy‐3‐methoxy flavone) and isorhamnetin‐3‐*O*‐β‐galactopyranoside were found (Abizov et al. 2008). Seven novel acylated isorhamnetin glycosides (elaeagnosides) A‐G (1–7) have also been isolated from the methanol extracts of *E. angustifolia* petals. Leaves were found to have higher total flavonoid contents than the fruits (Carradori et al. 2020).

### 4.4 Alkaloids

Different alkaloids were detected in roots, bark, and aerial parts. Elaeagnin or calligonin, which is structurally a tetrahydroharman, is among the most notable alkaloids obtained from *E. angustifolia* (Tehranizadeh et al. 2016). Also, 2‐methyl‐l,2,3,4‐tetrahydro‐β‐carboline, 1,2‐dihydroharmaline, dihydroharmane, 3,3‐dimethyl‐1,3‐dihydro‐indol‐2‐one, harmane, harmol, *N*‐methyl‐1,2,3,4‐tetrahydro‐β‐carboline, *N*‐methyltetrahydroharmol, tetrahydroharmine, tetrahydroharmol, and 2,3,4,9‐tetrahydro‐1‐methyl‐1H‐pyrido[3,4‐b] indole were identified from the bark of *E. angustifolia* (Tolkachev et al. 2008; Si et al. 2011).

### 4.5 Saponins and Tannins

Saponins and tannins have different biological activities including anthelmintic, hemostatic, antimicrobial, chemopreventive, angiogenesis, analgesic, cardioprotective, antitumor, and anti‐inflammatory activity (Motevalian et al. 2017). Variable concentrations of these compounds were observed in different organs of the *E. angustifolia* plant. Prominently, the fruit possesses significant amounts of saponins, whereas the maximum contents of tannins were found in bark, followed by the leaves and annual branches (Hamidpour et al. 2017).

### 4.6 Vitamins, Minerals, and Other Nutrients

*E. angustifolia* is recognized as a rich reservoir of different nutrients, including vitamins, minerals, sterols, steroids, alkanes, polyphenols (deca‐, undeca‐, and dodecaisoprenols), *trans*‐ethylcinnamate, cycloartenol, tocopherols, triterpene, aldehydes, citrostadienol, 24‐methylenecycloartanol, C21‐C31 hydrocarbons (mainly C29), C16‐C26 fatty alcohols, and α and β‐amyrins (Kiseleva and Chindyaeva 2011). Vitamins profile includes important vitamins, including vitamins A (provitamin A, β‐carotene), vitamin B, ascorbic acid (vitamin C), and vitamin K. Different minerals have also been found in *E. angustifolia* , with significant amounts of potassium, calcium, sodium, and phosphorous, and their abundance varies temporally and spatially (Abizov et al. 2008; Niknam et al. 2016).

## 5 Uses

### 5.1 Source of Food, Feed and Shelter for Wildlife, Domestic Animals and Humans

*E. angustifolia* is an excellent resource for both wildlife and humans alike as it provides feed, food, shelter (nest), windbreak, and controls soil erosion. Domestic animals, humans, birds, and honeybees feed on the branches, twigs, leaves, flowers, and fruits of *E. angustifolia* (Katz and Shafroth 2003).

### 5.2 Bio‐Fertilizer and Bio‐Monitor Activity

With the increasing population, the demand for food substantially enhanced, which demands the need to rehabilitate and recultivate contaminated and less fertile soils for sustainable agriculture. *E. angustifolia* is among those available stress‐tolerant plant species that can be efficiently used to cope with toxic heavy metals (Pb, Cd, Zn, Ni, and Hg) through bio‐adsorption. Furthermore, fruit powder of *E. angustifolia* acts as a good bio‐absorbent and symbiotically fix atmospheric nitrogen simultaneously (Follstad Shah et al. 2009). The leaves of *E. angustifolia* can also act as bio‐fertilizers because of the rich amount of nitrogen‐lignin contents (> 3%) and the ease of biodegradation (de Castro et al. 1990). Moreover, the presence of some allelopathic chemicals in the litter of *E. angustifolia* inhibits microbial nitrification; hence, it is very helpful in soil nitrogen availability. By fixing atmospheric nitrogen through symbiosis with Frankia bacteria, *E. angustifolia* improves soil fertility and greatly improves degraded croplands, even in nutrient‐poor and saline environments (Khamzina et al. 2009; Pretty Paint‐Small et al. 2013). The hardy nature of *E. angustifolia* and its adaptability across a wide range of soils in different geographical and ecological conditions, make it a potent bio‐fertilizer and bio‐monitor agent. Bio‐monitoring involves the ecological assessment of soil health and environmental interactions by using biological indicators such as soil microbe, vegetative dynamics, and insect pollinators and bio‐absorbent is the ability of plants to absorb heavy metals (Nistratov et al. 2020; Sardar et al. 2013).

### 5.3 Biological Insecticide

Increased use of synthetic insecticides which are mostly nonbiodegradable, poses a serious threat to the environment and human health. In this context, *E. angustifolia* offers a natural alternative. Extracts and fractions obtained from various parts of *E. angustifolia*, such as roots, leaves, flowers and fruits have shown promising insecticidal activity to control moth (*Ephestia cautella*) and different food grain storage pests including red flour beetle ( *Tribolium castaneum* ) (Khan et al. 2016; Niknam et al. 2016). Hence, *E. angustifolia* could also be used as a potential biological insecticide to control different insects of field and horticultural crops.

### 5.4 Medicinal Uses

**FIGURE 1:**
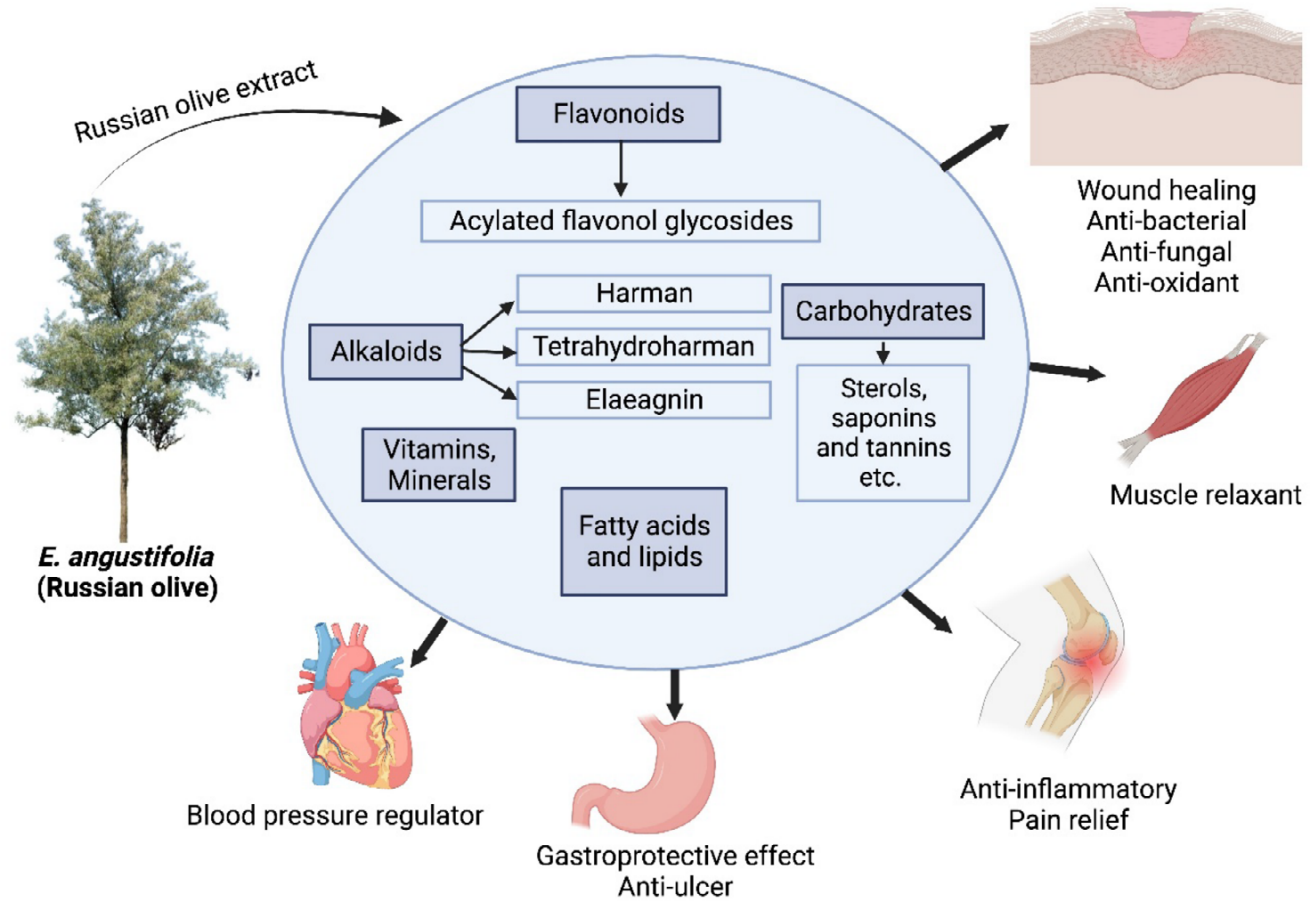
Schematic overview illustrating the various extracts derived from *Elaeagnus angustifolia* (Russian olive) and their associated medicinal importance.

Medicinal plants are a rich source of valuable natural compounds having various pharmaceutical properties. These include antioxidants, wound healing, analgesic and anti‐inflammatory applications in osteoarthritis and respiratory disorders, even demonstrating antimicrobial, antimutagenic, and anticancer properties, possessing cardiovascular and metabolic effects and helping in sustaining sexual health (Mohammadhosseini et al. 2019, 2021; Tehranizadeh et al. 2016). *E. angustifolia* and its constituents have long been used in the prevention or suppression of several diseases as summarized below (Figure 1).

#### 5.4.1 Analgesic and Anti‐Inflammatory Properties

**FIGURE 2:**
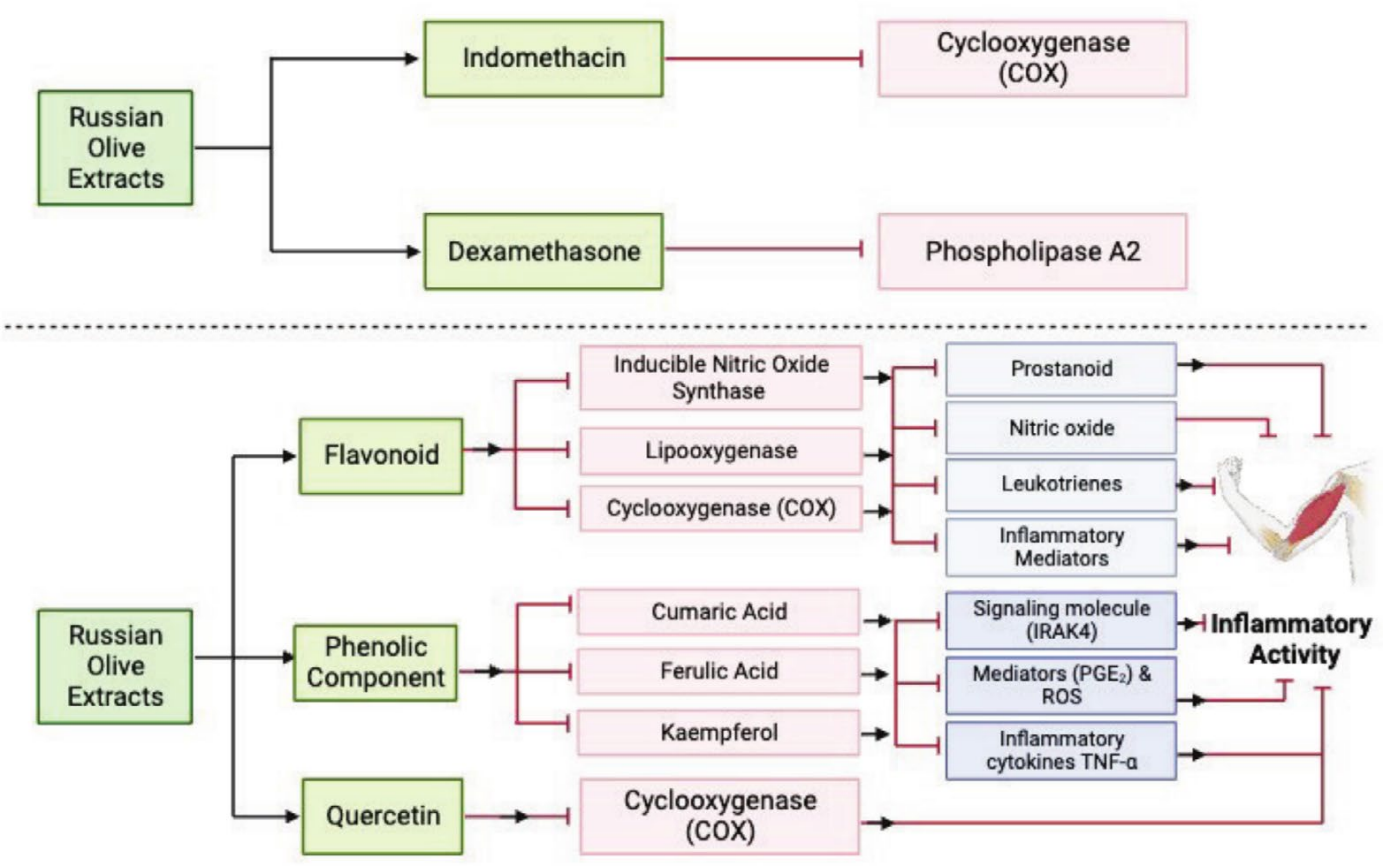
Proposed mechanism in alleviating muscular pain and inflammation by using *E. angustifolia* extracts (red lines show inhibition).

Polyphenols present in *E. angustifolia* such as cumaric acid, ferulic acid, and kaempferol have been reported to be involved in suppressing the release and production of inflammatory mediators (COX‐1, COX‐2, PGE2 and nitric oxide), signaling molecules (IRAK4, Src, and Syk), inflammatory cytokines (TNF‐α and interleukin‐6) and ROS production (Farahbakhsh et al. 2011). In addition, Motevalian et al. (2017) observed that *E. angustifolia* fruit extract possesses acute and chronic anti‐inflammatory activities in rat paw edema through diverse mechanisms (Figure 2). Similarly, Ahmadiani et al. (2000) also reported the anti‐nociceptive and anti‐inflammatory activities of *E. angustifolia* fruit extract.

#### 5.4.2 Osteoarthritis

The pain‐relieving and muscle‐relaxant properties of *E. angustifolia* extracts provide relief to patients with osteoarthritis. However, it is important to note that long‐term muscle relaxation is considered harmful because strengthening of quadriceps muscles is required, along with muscle relaxation for osteoarthritis treatment (Hosseinzadeh et al. 2003; Panahi et al. 2016). Studies have shown that fruit extracts of *E. angustifolia* have efficacy in alleviating osteoarthritic symptoms. A water extract derived from fruit was shown to reduce serum matrix metalloproteinase 3 and 13 and pathological signs in mice with monosodium iodoacetate‐induced osteoarthritis (Heydari Nasrabadi et al. 2022).

More convincingly, Nikniaz et al. (2014) demonstrated that the administration of 15 g/day of *E. angustifolia* medulla and whole fruit powders to women affected by knee osteoarthritis resulted in a decrease of matrix metalloproteinase‐1, TNF‐α and an increase of the anti‐inflammatory cytokine IL‐10. These data are consistent with the work of Karimifar et al. (2017) who observed the administration of an *E. angustifolia* fruit water extract, at a daily dose of 200 mg, for 4 weeks, to knee osteoarthritis affected patients of 40–80 years old, resulted in an improvement of visual analog scale (VAS), Lequesne pain and function index (LPFI) and patient global assessment (PGA). Also, osteoarthritic patients 50–80 years old treated with a daily dose of 300 and 400 mg of an *E. angustifolia* fruit water extract for 7 weeks experienced improvements measured as Western Ontario and McMaster Universities Osteoarthritis Index, VAS, LPFI, and PGA index. This study identified the alkaloids (Harmine and Harmaline) and polyphenols in fruit extracts that reduce oxidative stress (Panahi et al. 2016; Nikniaz et al. 2014).

#### 5.4.3 Wound Healing

*E. angustifolia* extracts contain different chemicals in the highest concentrations, such as vitamins A, B, K, and E, which are important for wound healing (Natanzi et al. 2012; Esmaeili and Niknam 2013; Hamidpour et al. 2019). Natanzi et al. (2012) demonstrate that the water fruit extracts of *E. angustifolia* improve the hydroxyproline content, wound healing, and histological score of rats with skin wounds. Also, the flower's soft extract derived from *E. angustifolia* (cream‐based) showed a wound healing effect on rabbits with scarred skin. Extracts have been reported to significantly impact all the phases of the wound healing process including hemostasis, inflammatory response, proliferation, and tissue repair (Bucur et al. 2008).

#### 5.4.4 Antimicrobial Activity

*E. angustifolia* extracts derived from its leaves, flowers and fruits have shown significant antimicrobial activity both against bacteria and fungi. Methanolic phyto‐complex from flowers, leaves and fruits showed antimicrobial activities against several bacteria with some differences. Particularly, flower extract was the most active against gram‐positive bacteria ( *Bacillus cereus* , *Staphylococcus aureus* , *Staphylococcus epidermidis* and *Enterococcus faecalis* ), followed by leaves and fruit extracts (Incilay 2014). This difference likely reflects the higher amount of volatile compounds (terpenes, alcohols, aldehydes, ketones and esters) in flowers than in leaves and fruits. These volatile compounds are endowed with antimicrobial effects.

Methanolic extract of the whole plant and its fractions of increasing polarity were tested against several pathogenic bacteria and fungi. The methanolic extract of the whole plant and the water fraction had no effects on *Pseudomonas aeruginosa* . The water fraction had the highest potency against *Escherichia coli* , while the ethylacetate fraction was the most active against *S. aureus* and *P. aeruginosa* . No antibacterial effects were observed with the chloroform fraction. The crude extract was active against *Aspergillus fumagatus* but inactive against *Aspergillus flavis* and *Aspergillus niger*. The water fraction inhibited only *A. flavis*; the ethylacetate fraction was active against *A. flavis* and *A. niger* , without any effects on *A. fumagatus*. The chloroform fraction had no activity, while the *n*‐hexane fraction was the most active against all the tested strains (Khan et al. 2016). The antimicrobial activities of a methanolic extract obtained from *E. angustifolia* L. leaves were demonstrated for *Bacillus subtilis* , *Staphylococcus aureus* , *Listeria monocytogenes* , *Enterococcus faecalis* , *Yersinia enterocolitica* , *Salmonella typhimurium* , with the highest effect on *Yersinia enterocolitica* (Okmen and Turkcan 2014). Therefore, it can be effectively used for preventing and treating several bacterial and fungal diseases including mastitis in animals and dental hygiene in humans (Incilay 2014).

#### 5.4.5 Respiratory Disorders

Folk medicine has long employed decoction and infusion made from the bark, leaf, flower, and fruit to treat respiratory conditions. Beyond documented antimicrobial activities of *E. angustifolia* , this plant also exhibited notable anti‐inflammatory and antioxidant effects, further justifying its potential as a nutraceutical against respiratory diseases. Specifically, extracts of *E. angustifolia* can be effectively used in the treatment of excessive mucus production, airway hyper‐responsiveness, shortness of breath, sore throat, flu, cold, fever, cough, and wheezing (Ge et al. 2009). This traditional use of *E. angustifolia* is further substantiated by a recent study by Mamashli et al. (2022). He observed that *E. angustifolia* fruit water extract exerts protective effects toward carrageenan‐induced acute lung injury through several mechanisms involving the decrease of IL‐6, TNF‐α and oxidative stress.

**FIGURE 3:**
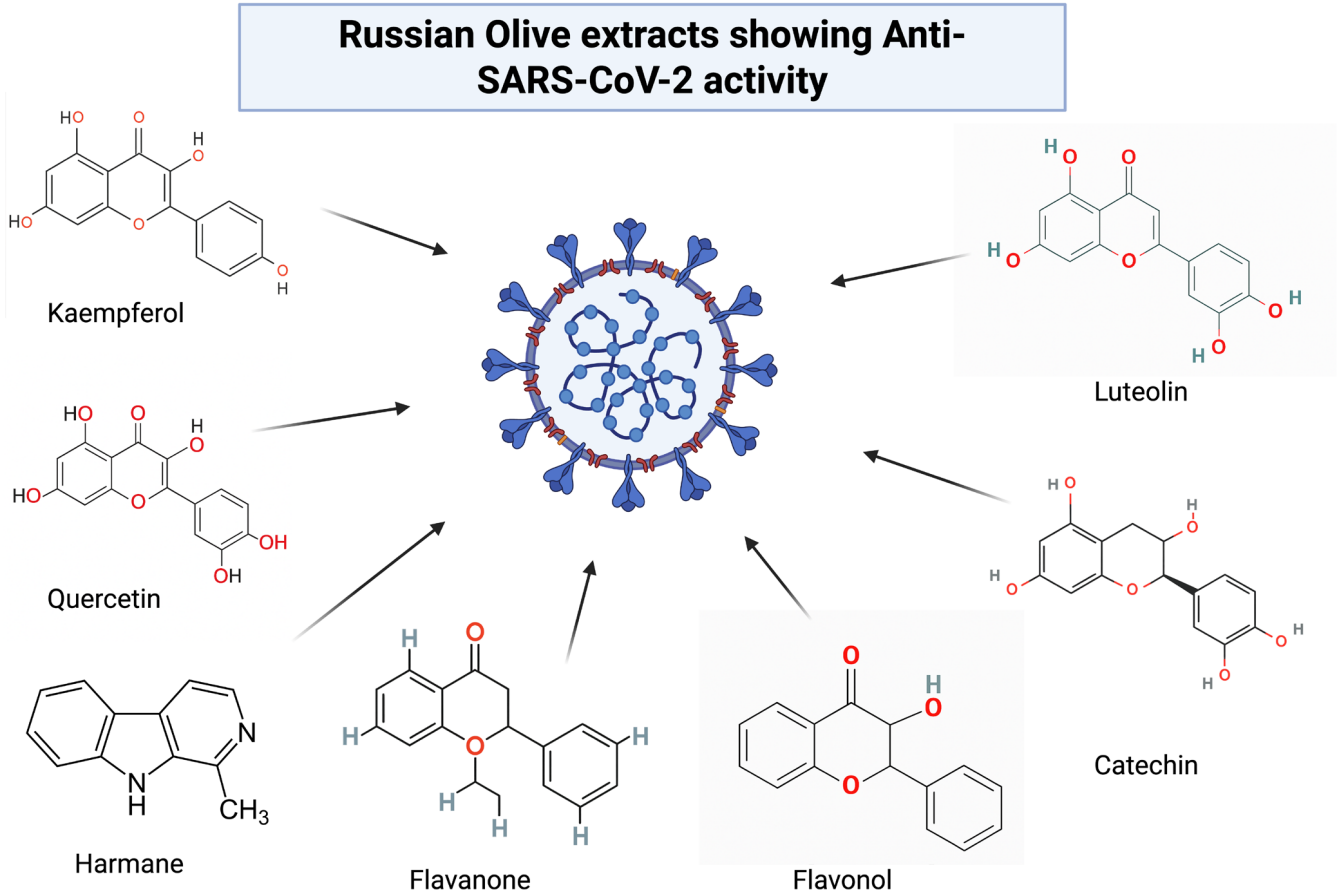
Schematic illustration depicting various components of *E. angustifolia* and their proposed mechanism of action against SARS‐CoV‐2.

Moreover, *E. angustifolia* have potential biochemicals such as kaempferol, quercetin, flavanone, flavanol, catechin, luteolin, harmane, etc. that assist in curing emerging diseases, for instance, COVID‐19 (as shown in Table 2). Several studies demonstrated the anti‐inflammatory, and immune‐modulatory properties of these chemicals in coping with COVID‐19 (Figure 3) (Anand et al. 2021; Ang et al. 2020; Boukhatem and Setzer 2020; Chakravarti et al. 2021; Chojnacka et al. 2020; Mazraedoost et al. 2021; Nile et al. 2021; Sohail et al. 2021; Srivastava et al. 2020; Wink 2020). However, clinical studies are needed to validate the efficacy of *E. angustifolia* extracts in coping with COVID‐19, standardize the appropriate dosage, and elucidate the mechanism of action.

**TABLE 2:** Summary of various plant extracts, their antiviral properties, and proposed possible mechanisms of therapeutic action.

Plant	Chemical (extracts)	Active against	Mechanism of action	References
Natural herbs, fruit and vegetables	Flavanone, flavanol, triterpenes, quinone and curcumin derivatives	Anti‐SARS‐CoV‐2 activity	Bind with 3 chymotrypsin‐like protease (3CLpro), spike protein (TMPRSS2), and angiotensin‐converting enzyme 2 (ACE2), ultimately inhibit COVID‐19 inoculation and replication in host cell	da Silva Antonio et al. (2020)
Natural plants	Natural extracts like flavonoids, terpenoids, diarylheptanoids, and coumarins	Anti‐SARS‐CoV‐2 activity	Effective inhibitors of the SARS‐CoV proteases	Chakravarti et al. (2021)
*Dysphania ambrosioides* (Mexican‐tea)	Flavonoid glycoside like rutin and nicotiflorin	Anti‐SARS‐CoV‐2 activity	Effectively inhibit the SARS‐CoV‐2 RNA‐dependent RNA polymerase (RdRp) and SARS‐CoV‐2 main protease (M^PRO^), thus inhibiting the viral life cycle inside host	Chakravarti et al. (2021)
Red onion ( *Allium cepa* )	Flavonoid content as Quercetin	Anti‐SARS‐CoV‐2 activity	Effectively inhibit the viral life cycle and entry in host cell, by binding with SARS‐CoV M^PRO^ and SARS‐CoV Spike (S) protein	Chakravarti et al. (2021), Srivastava et al. (2020)
Natural plants	Flavonol such as Kaempferol	Anti‐SARS‐CoV‐2 activity	Specifically bind to Spike glycoproteins of coronavirus and Inhibit the papain‐like proteases (PL^PRO^), 3CLpro and CoV M^PRO^	Anand et al. (2021), Nile et al. (2021), Sohail et al. (2021)
*Broussonetia papyrifera*	Polyphenols	Anti‐SARS‐CoV activity	Inhibition of CoV cysteine proteases	Chojnacka et al. (2020)
*Curcuma* sp.	Polyphenols like curcumin and its derivatives	Anti‐SARS‐CoV‐2 activity	Target the MERS‐CoV 3CLpro, hence preventing the viral entry in host cell	Chakravarti et al. (2021), Mazraedoost et al. (2021)
Giloy (*Tinospora cordifolia*)	Sitosterol	Anti‐SARS‐CoV‐2 activity	Exhibiting strong anti‐COVID properties by binding with COVID‐19 protease	Srivastava et al. (2020)
Natural plants	Vitamin C (ascorbic acid)	Anti‐SARS‐CoV‐2 activity	Immunomodulator agent and antioxidant property	Nile et al. (2021)
Herbs	Luteolin	Antiviral activity	Specifically bind and interact with S2 domain of SARS‐CoV‐2 S proteins, and inhibiting the entry of virus (HIV‐luc/SARS pseudo‐type virus)	Boukhatem and Setzer (2020), Sohail et al. (2021)
*Euphorbia hirta* L.	Tannins extracts	Strong antiretroviral activity	Inhibit viral replication	Sytar et al. (2021)
*Anagallis arvensis*	Saponin	Antiviral activity against HSV‐1, poliovirus‐2 and SARS‐CoV	Inhibit virion to attach, enter, absorb and penetrate into the host cell, also protect the host cell from structural injury	Anand et al. (2021)
Green tea	Catechin	Therapeutic agent for COVID‐19	Bind with ACE2 of the host and receptor domains of viral S‐protein	Anand et al. (2021)
*O. sanctum*	Luteolin	Anti‐SARS–CoV property and most potent against coxsackievirus A16 and enterovirus‐71 infections	Bind with human receptor, and inhibiting the entry of SARS–CoV, as well as it disrupts viral RNA replication	Anand et al. (2021), Chakravarti et al. (2021)
*Zygophyllaceae*	β‐Carboline alkaloids (harmine, harmaline)	Anti HSV, MCMV, influenza	DNA intercalation, inhibition of topoisomerase, DNA polymerase, reverse transcriptase	Wink (2020)
*Allium cepa* L.	Linolenic acid, palmitic acid, oleanolic acid	Anti‐SARS‐CoV‐2 activity	Target specifically M^PRO^ and 3CLpro	Kim (2021)
Medicinal plants	Terpenoids	Antiviral activity	Inhibit the activity of protease in viruses, by hampering specific amino acid	Sohail et al. (2021)

#### 5.4.6 Sexual Health

The extracts from *E. angustifolia* flowers have shown effectiveness in recovering sexual function in females by widening blood vessels, decreasing swelling in the genital system, and enhancing the relaxation of smooth vaginal muscles (Akbarzadeh et al. 2014). Furthermore, a clinical trial suggested the efficacy of this plant in improving sexual interest/arousal in women (Zeinalzadeh et al. 2019). Furthermore, studies show mild estrogenic activity in menopausal women (Emaminia et al. 2020; Jalalvand et al. 2021).

#### 5.4.7 Antioxidant Effect

The experiment of power assays, DPPH (2,2′‐diphenyl‐1‐picrylhydrazyl radical), and FRAP (fluorescence recovery after photobleaching) assays confirm that the methanolic extracts derived from the seeds and peels of *E. angustifolia* showed the antioxidant property. The seed extracts have a higher amount of flavonoids and phenolic compounds than the peels and flesh. Therefore, seed extracts have the highest antioxidant activity (Faramarz et al. 2015). Yalcin and Sogut (2014) experiment showed that the ethanol/water extracts present in the peel, leaves, mesocarp, and their mixture also exhibit higher antioxidant properties compared to the methanol/water and ethanol/acetone extracts.

Sarvarian et al. (2022) observed that the *E. angustifolia* extracts added to the orange juice increase the antioxidant capacity. In addition, crackers prepared with *E. angustifolia* L. fruit flour have higher polyphenol amounts and antioxidant effects than the control foods (Incedayi and Erol 2023). Likewise, its fruit flour is added to ice creams, which improves the chemical, sensory, and physical properties along with boosting antioxidant capacity (Çakmakçı et al. 2015). Thus, data suggest that the *E. angustifolia* extracts are used for nutraceutical products.

#### 5.4.8 Antimutagenic and Anticancer Activity

*E. angustifolia* contains methanolic extracts that have antioxidant and anti‐inflammatory properties, thus serve as antitumor and antimutagenic agents (Amereh et al. 2017). In 2014, Okmen and Turkcan used the Ames test in the absence of rat microsomal liver enzyme (‐S9) and confirmed that the methanolic extracts of *E. angustifolia* positively inhibit the mutagenic activities (Okmen and Turkcan 2014).

An *E. angustifolia* water extract (EAE) has demonstrated selective cytotoxic activity against cancer cells by inhibiting MDA‐MB‐231 and MDA‐MB‐436 proliferation while minimal affecting nontumorigenic epithelial cell line, MCF 10A. The phyto‐complex acts on these cancer cells through the induction of early and late apoptosis as evidenced by an increase of pro‐apoptotic markers (Bax and cleaved caspase‐8) and a decrease in the antiapoptotic protein Bcl2. Furthermore, it is found that EAE inhibits cancer cell colony formation partly due to the upregulation of p53 and the inhibition of signal transducer and activator of transcription 3 (STAT3) phosphorylation (Fouzat 2021).

In another study, the similar EAE extract inhibited the antiproliferating activity and colony formation of Human Epidermal Receptor 2 (HER2)‐positive breast cancer, Sloan‐Kettering Institute's Breast Cancer 3 (SKBR3, breast cancer cell line) and ZR75‐1 cell (human breast cancer cell line) by modulating mesenchymal–epithelial transition. Specifically, EAE extracts activate the E‐cadherin and β‐catenin upregulation, vimentin, p‐β‐catenin, and fascin downregulation. Thereby promoting epithelial cell development. Additionally, EAE modulates the deregulation of JNK1/2 (c‐Jun N‐terminal Kinase 1 and 2) and promotes E‐cadherin and β‐catenin expression while maintaining reduced expression of vimentin and fascin (Jabeen et al. 2020). Apart from breast cancer, EAE extract also possesses anticancer effects when observed in oral carcinoma cell lines such as FaDu and SCC25. Indeed, the phyto‐complex inhibited proliferation and induced apoptosis in addition to upregulating E‐cadherin expression and inhibiting angiogenesis (Saleh et al. 2018).

Additionally, it was stated that *E. angustifolia* fruit extract prevented colorectal cancer in *Drosophila melanogaster* with a KRAS (Kirsten Rat Sarcoma viral oncogene homolog) gene mutation (Fouzat 2021; Zakaria et al. 2023). The chemopreventive potential of this plant was investigated also for fruit extracts. Indeed, in vivo data showed the ability of an *E. angustifolia* fruit extract to inhibit diethyl nitrosamine‐induced hepatocarcinogenesis in rats through several mechanisms involving inflammation and oxidative stress to prevent colorectal cancer cells (Amereh et al. 2017).

#### 5.4.9 Cardiovascular and Metabolic Effects

**FIGURE 4:**
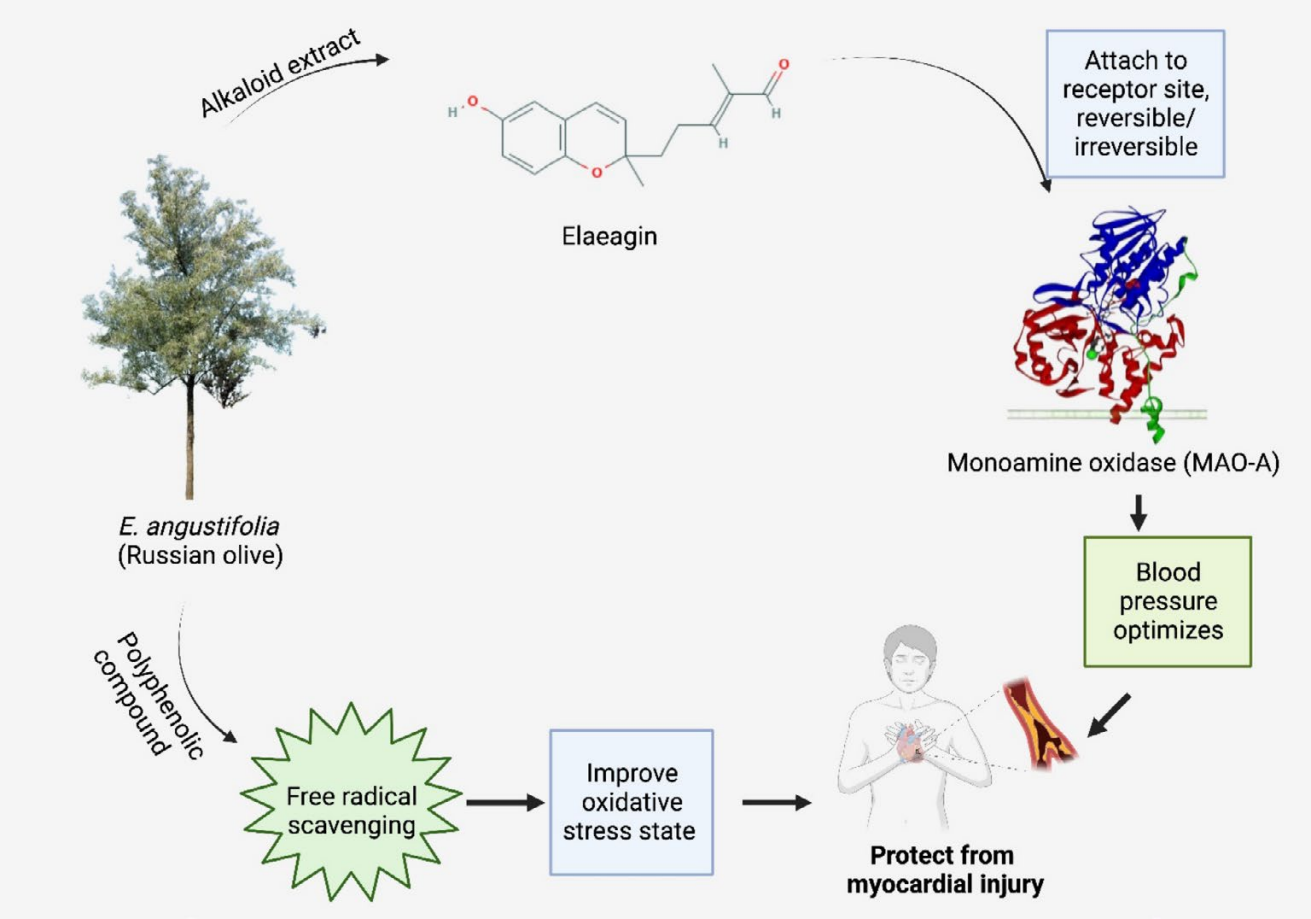
*E. angustifolia* extract is involved in the healing of cardiovascular injury by its free radical scavenging and myocardial protective activity.

Antioxidant, free radical scavenging, anti‐inflammatory, and wound healing properties of *E. angustifolia* extracts make them a potential treatment for cardiovascular diseases (acute myocardial infarction and myocardial reperfusion injury) (Okmen and Turkcan 2014). A leaf extract of *E. angustifolia* has shown a protective effect against myocardial ischemia/reperfusion injury due to the presence of different phytochemicals binding to mono‐amino oxidase A (MAO‐A) active site in humans (Tehranizadeh et al. 2016). The ability of *E. angustifolia* extracts to inhibit oxidative stress both directly and indirectly through the increase of superoxide dismutase (SOD) activity contributes to safeguarding cardiac tissues and maintaining cardiac functions compromised by ischemia/reperfusion (Wang et al. 2014). The detailed illustrations involving the mechanism of healing during cardiovascular injury by using *E. angustifolia* extracts are shown in Figure 4.

Additionally, the consumption of whole fruit powder of *E. angustifolia* appears beneficial for cardiovascular health. A clinical study showed that the daily administration of 15 g of this substance for 10 weeks in postmenopausal women resulted in a decrease in low‐density lipoprotein (LDL) cholesterol (Shabani et al. 2021). Furthermore, the administration of 15 g/day of *E. angustifolia* medulla powder or whole fruit powder for 8 weeks to obese females was reported to reduce total cholesterol, TC/HDL, and LDL/HDL ratios (Nikniaz et al. 2016). Similarly, Belarbi et al. (2011) reported that the administration of *E. angustifolia* favorably affects total cholesterol, LDL‐cholesterol, triglycerides, and HDL‐cholesterol in healthy individuals.

These reported beneficial effects of *E. angustifolia* are mainly attributed due to the presence of bioactive compounds including, polyphenols and alkaloids. In particular, harmine and harmaline alkaloids found in this plant were demonstrated to reduce total cholesterol in rats favoring cholesterol 7 alpha‐hydroxylase activity (Vahidi‐eyrisofla et al. 2015).

#### 5.4.10 Gastrointestinal Effects

The *E. angustifolia* contains the carotenoid and fruit methanolic content that has the ulcer‐protecting capability (Tehranizadeh et al. 2016). Its extract also has the relaxing property of lumen muscle contraction through the antioxidant pathways, which also act as a barrier to protect the stomach surface from gastric mucosa (Tehranizadeh et al. 2016). In Armenia, the pshatin drug has been derived from the fruit extract of *E. angustifolia* (specifically polyphenolic compound), which effectively acts against colitis and gastrointestinal tract diseases. Their lead and fruit extracts are effective in treating nausea, flatulence, and vomiting (Hamidpour et al. 2019, 2017).

## 6 Side Effects

Study‐based analysis concludes no detrimental side effects of this plant such as induction of abnormalities or delayed growth, even of excessive usage of extracts, but the pollens of this plant act like allergens and may trigger nasal irritation symptoms (Hamidpour et al. 2017). On the contrary, it is observed that the potent toxicity of *E. angustifolia* is due to the presence of the high amount of E‐ethyl cinnamate in the essential oil of their leaves and flowers, showing notable antifeeding, insecticidal and nematocidal activities. However, the high amount of E‐ethyl cinnamate does not cause any noticeable side effects in mammals or humans (Torbati et al. 2016). The aqueous and ethanol extract of *E. angustifolia* has muscle‐relaxant properties, however, long‐term use might be harmful because treatment of osteoarthritis requires quadriceps muscle strengthening (Ebrahimi et al. 2014; Panahi et al. 2016). Additionally, studies confirm that the aqueous extract of *E. angustifolia* fruit in pregnant mice was safe and nonpathogenic (Farzaei et al. 2015).

Notably, the extract of *E. angustifolia* may reduce the side effects and increase the effectiveness of some chemotherapy medications, for instance, cisplatin (Zakaria et al. 2023). Research has demonstrated that the fruit extract *E. angustifolia* has cytotoxic and antiproliferation potency on U87 and C6 (human and rat) glioblastoma cancer cells, thus considered a safe pharmaceutical plant to treat glioblastoma cancer (Arab et al. 2022) with no toxic or very minimal effects on normal body cells (Zakaria et al. 2023). However, the anticancer property of *E. angustifolia* depends upon the type and concentration of extract used (Arab et al. 2022). Therefore, more comprehensive clinical studies are required to find out the potential side effects, toxicity, optimal dosage recommendation to use by patients, and long‐term safety of *E. angustifolia* extracts.

## 7 Conclusion and Future Perspective

*E. angustifolia* holds significant potential in improving human health as the different parts are rich sources of different bioactive compounds including flavonoids, phenolic acids, tannins, alkaloids, polysaccharides, water and fat‐soluble vitamins, minerals, and lipids. Its potential for incorporation into food matrices such as fruit juices, bakery products, and ice creams offers the possibility of developing functional foods with aided health benefits. However, standardizing extraction processes, determining optimal harvesting times, and establishing quality control measures can be challenging in ensuring product consistency and efficacy. Further, cost‐effective extraction methods and efficient processing techniques are essential for the economic viability of valorizing *E. angustifolia* . Preclinical and clinical studies suggest that this plant could be a resource for the development of nutraceuticals useful in the management of pathological conditions characterized by inflammation and oxidative stress, particularly in cardiovascular and osteoarticular diseases. Nonetheless, there is a need to determine the optimal dosage of *E. angustifolia* nutraceuticals for therapeutic effects while avoiding potential side effects. Additionally, preliminary data suggest a possible application of *E. angustifolia* phyto‐complexes in the cosmeceutical field. However, rigorous scientific studies are needed to establish the efficacy and safety of *E. angustifolia* phyto‐complexes in cosmeceutical applications. Future research should also focus on exploring the functions of *E. angustifolia* extracts in combating emerging diseases, such as COVID‐19 as it has an active phytochemical profile. Furthermore, well‐designed clinical studies are required to evaluate various extract concentrations in managing different diseases and exploring the underlying mechanisms.

## Author Contributions

**Muhammad Abubakkar Azmat:** conceptualization (equal), writing – original draft (equal), writing – review and editing (equal). **Aqeel Shahzad:** conceptualization (equal), writing – original draft (equal), writing – review and editing (equal). **Muhammad Shaban:** supervision (equal), validation (equal), visualization (equal), writing – review and editing (equal). **Asif Ali Khan:** writing – review and editing (equal). **Sadaf Shakoor:** writing – review and editing (equal).

## Ethics Statement

The authors have nothing to report.

## Conflicts of Interest

The authors declare no conflicts of interest.

## Data Availability

Data sharing not applicable to this article as no datasets were generated or analyzed during the current study.

## References

[fsn370486-bib-0001] Abdalla, T. E. 2019. “Some Wild *Elaeagnus* Species: Overview, Description, Biochemistry, and Utilization.” In Wild Fruits: Composition, Nutritional Value Products, 507–521. Springer Nature.

[fsn370486-bib-0002] Abizov, E. , O. Tolkachev , S. Mal'Tsev , and E. Abizova . 2008. “Composition of Biologically Active Substances Isolated From the Fruits of Russian Olive ( *Elaeagnus angustifolia* ) Introduced in the European Part of Russia.” Pharmaceutical Chemistry Journal 42, no. 12: 696–698.

[fsn370486-bib-0003] Ahmadiani, A. , J. Hosseiny , S. Semnanian , et al. 2000. “Antinociceptive and Anti‐Inflammatory Effects of *Elaeagnus angustifolia* Fruit Extract.” Journal of Ethnopharmacology 72, no. 1–2: 287–292. 10.1016/s0378-8741(00)00222-1. 10967484

[fsn370486-bib-0004] Akbarzadeh, M. , S. Zeinalzadeh , J. Zolghadri , A. Mohagheghzadeh , P. Faridi , and M. Sayadi . 2014. “Comparison of *Elaeagnus angustifolia* Extract and Sildenafil Citrate on Female Orgasmic Disorders: A Randomized Clinical Trial.” Journal of Reproduction & Infertility 15, no. 4: 190–198. 25473627 PMC4227976

[fsn370486-bib-0005] Amereh, Z. , N. Hatami , F. H. Shirazi , et al. 2017. “Cancer Chemoprevention by Oleaster (*Elaeagnus angustifoli* L.) Fruit Extract in a Model of Hepatocellular Carcinoma Induced by Diethylnitrosamine in Rats.” EXCLI Journal 16: 1046–1056. 10.17179/excli2017-389. 28900384 PMC5579409

[fsn370486-bib-0006] Anand, A. V. , B. Balamuralikrishnan , M. Kaviya , et al. 2021. “Medicinal Plants, Phytochemicals, and Herbs to Combat Viral Pathogens Including SARS‐CoV‐2.” Molecules 26, no. 6: 1775. 10.3390/molecules26061775. 33809963 PMC8004635

[fsn370486-bib-0007] Ang, L. , E. Song , H. W. Lee , and M. S. Lee . 2020. “Herbal Medicine for the Treatment of Coronavirus Disease 2019 (COVID‐19): A Systematic Review and Meta‐Analysis of Randomized Controlled Trials.” Journal of Clinical Medicine 9, no. 5: 1583. 10.3390/jcm9051583. 32456123 PMC7290825

[fsn370486-bib-0008] Antunes, L. L. , A. L. Back , M. Kossar , A. G. Spessato , E. Colla , and D. A. Drunkler . 2023. “Prebiotic Potential of Carbohydrates From Defatted Rice Bran – Effect of Physical Extraction Methods.” Food Chemistry 404, no. Pt A: 134539. 10.1016/j.foodchem.2022.134539. 36242965

[fsn370486-bib-0009] Arab, S. , M. Bahraminasab , A. Yazdani , and A. Abdolshahi . 2022. “Effects of Whole Fruit Extract of *Elaeagnus angustifolia* L. on Glioblastoma Cell Lines.” Journal of Microbiology, Biotechnology and Food Sciences 11, no. 5: e4314.

[fsn370486-bib-0010] Arroo, R. R. J. , K. Beresford , A. S. Bhambra , et al. 2014. “Phytoestrogens as Natural Prodrugs in Cancer Prevention: Towards a Mechanistic Model.” Phytochemistry Reviews 13: 853–866.

[fsn370486-bib-0011] Asadiar, L. S. , F. Rahmani , and A. Siami . 2013. “Assessment of Genetic Diversity in the Russian Olive ( *Elaeagnus angustifolia* ) Based on ISSR Genetic Markers.” Revista Ciência Agronômica 44: 310–316.

[fsn370486-bib-0012] Ayaz, F. A. , and E. Bertoft . 2001. “Sugar and Phenolic Acid Composition of Stored Commercial Oleaster Fruits.” Journal of Food Composition and Analysis 14, no. 5: 505–511.

[fsn370486-bib-0013] Ayaz, M. , A. Sadiq , M. Junaid , et al. 2019. “Flavonoids as Prospective Neuroprotectants and Their Therapeutic Propensity in Aging Associated Neurological Disorders.” Frontiers in Aging Neuroscience 11: 155. 31293414 10.3389/fnagi.2019.00155 PMC6606780

[fsn370486-bib-0014] Azmat, M. A. , A. A. Khan , I. A. Khan , A. Buerkert , and M. Wiehle . 2020. “Morphology, Biochemistry, and Management of Russian Olive ( *Elaeagnus angustifolia* L.) Accessions in Gilgit‐Baltistan, Northern Pakistan.” Journal of Agriculture and Rural Development in the Tropics and Subtropics 121, no. 2: 151–160.

[fsn370486-bib-0015] Bartish, I. V. , N. Jeppsson , H. Nybom , and U. Swenson . 2002. “Phylogeny of Hippophae (Elaeagnaceae) Inferred From Parsimony Analysis of Chloroplast DNA and Morphology.” Systematic Botany 27, no. 1: 41–54.

[fsn370486-bib-0016] Bartish, I. V. , and U. Swenson . 2004. “Elaeagnaceae.” In Flowering Plants· Dicotyledons: Celastrales, Oxalidales, Rosales, Cornales, Ericales, 131–134. Springer.

[fsn370486-bib-0017] Bekker, N. P. , and A. I. Glushenkova . 1997. “Lipids of the Leaves of *Elaeagnus angustifolia* . I. Surface Lipids.” Chemistry of Natural Compounds 33: 543–544.

[fsn370486-bib-0018] Belarbi, M. , S. Bendimerad , S. Sour , et al. 2011. “Oleaster Oil Positively Modulates Plasma Lipids in Humans.” Journal of Agricultural and Food Chemistry 59, no. 16: 8667–8669. 10.1021/jf201865z. 21761860

[fsn370486-bib-0019] Bonner, F. T. 2008. The Woody Plant Seed Manual. Forest Service.

[fsn370486-bib-0020] Boukhatem, M. N. , and W. N. Setzer . 2020. “Aromatic Herbs, Medicinal Plant‐Derived Essential Oils, and Phytochemical Extracts as Potential Therapies for Coronaviruses: Future Perspectives.” Plants 9, no. 6: 800. 32604842 10.3390/plants9060800 PMC7356962

[fsn370486-bib-0021] Brock, J. H. 2003. “ *Elaeagnus angustifolia* (Russian Olive) Seed Banks From Invaded Riparian Habitats in Northeastern Arizona.” In Plant Invasions: Ecological Threats Management Solutions, 267–276. Backhuys Publishers.

[fsn370486-bib-0022] Bucur, L. , G. Taralunga , R. Alexandrescu , T. Negreanu , and V. Istudor . 2008. “Evaluation of Biological Activity of a Dermatological Preparation With *elaeagnus angustifolia* Flowers Soft Extract.” Revista Medico‐Chirurgicală̆ a Societă̆ţii de Medici ş̧i Naturaliş̧ti din Iaş̧i 112, no. 4: 1098–1103. 20209794

[fsn370486-bib-0023] Çakmakçı, S. , E. F. Topdaş , P. Kalın , et al. 2015. “Antioxidant Capacity and Functionality of Oleaster (*Elaeagnus angustifolia* L.) Flour and Crust in a New Kind of Fruity Ice Cream.” International Journal of Food Science & Technology 50, no. 2: 472–481.

[fsn370486-bib-0024] Carradori, S. , F. Cairone , S. Garzoli , et al. 2020. “Phytocomplex Characterization and Biological Evaluation of Powdered Fruits and Leaves From *Elaeagnus angustifolia* .” Molecules 25, no. 9: 2021. 10.3390/molecules25092021. 32357533 PMC7248930

[fsn370486-bib-0025] Chakravarti, R. , R. Singh , A. Ghosh , et al. 2021. “A Review on Potential of Natural Products in the Management of COVID‐19.” RSC Advances 11, no. 27: 16711–16735. 10.1039/d1ra00644d. 35479175 PMC9031656

[fsn370486-bib-0026] Chen, Q. , J. Chen , H. Du , et al. 2014. “Structural Characterization and Antioxidant Activities of Polysaccharides Extracted From the Pulp of *Elaeagnus angustifolia* L.” International Journal of Molecular Sciences 15, no. 7: 11446–11455. 10.3390/ijms150711446. 24972139 PMC4139792

[fsn370486-bib-0027] Chojnacka, K. , A. Witek‐Krowiak , D. Skrzypczak , K. Mikula , and P. Młynarz . 2020. “Phytochemicals Containing Biologically Active Polyphenols as an Effective Agent Against Covid‐19‐Inducing Coronavirus.” Journal of Functional Foods 73: 104146. 32834835 10.1016/j.jff.2020.104146 PMC7392194

[fsn370486-bib-0028] da Silva Antonio, A. , L. S. M. Wiedemann , and V. F. Veiga‐Junior . 2020. “Natural Products' Role Against COVID‐19.” RSC Advances 10, no. 39: 23379–23393. 35693131 10.1039/d0ra03774e PMC9122563

[fsn370486-bib-0029] de Castro, F. B. , Y. Aranda , and M. F. Schmitz . 1990. “Acetylene‐Reducing Activity and Nitrogen Inputs in a Bluff of *Eleagnus angustifolia* L.” Orsis: Organismes i Sistemes 5: 85–89.

[fsn370486-bib-0030] Du, H. , J. Chen , S. Tian , et al. 2016. “Extraction Optimization, Preliminary Characterization and Immunological Activities In Vitro of Polysaccharides From *Elaeagnus angustifolia* L. Pulp.” Carbohydrate Polymers 151: 348–357. 10.1016/j.carbpol.2016.05.068. 27474576

[fsn370486-bib-0031] Ebrahimi, A. A. , Z. Nikniaz , A. Ostadrahimi , R. Mahdavi , and L. Nikniaz . 2014. “The Effect of *Elaeagnus angustifolia* L. Whole Fruit and Medulla Powder on Women With Osteoarthritis of the Knee: A Randomized Controlled Clinical Trial.” European Journal of Integrative Medicine 6, no. 6: 672–679. 10.1016/j.eujim.2014.07.016.

[fsn370486-bib-0032] Emaminia, F. , A. Rezaei , B. Badehnoosh , R. Ramezani , and M. Shabani . 2020. “The Effects of *Elaeagnus angustifolia* L. Whole Fruit on the Sex Hormone Profile in Menopausal Women: A Double‐Blind, Randomized, Placebo‐Controlled Study.” Journal of Ethnopharmacology 246: 112229. 10.1016/j.jep.2019.112229. 31513839

[fsn370486-bib-0033] Enescu, C. M. 2018. “Russian Olive ( *Elaeagnus angustifolia* L.): A Multipurpose Species With an Important Role in Land Reclamation.” Current Trends in Natural Sciences 7, no. 13: 54–60.

[fsn370486-bib-0034] Ersoy, N. , I. H. Kalyoncu , A. Y. Elidemir , and I. Tolay . 2013. “Some Physico‐Chemical and Nutritional Properties of Russion Olive ( *Elaeagnus angustifolia* L.) Fruit Grown in Turkey.” International Journal of Agricultural Biosystems Engineering 7, no. 6: 427–429.

[fsn370486-bib-0035] Esmaeili, A. , and S. Niknam . 2013. “Characterization of Nanocapsules Containing *Elaeagnus angustifolia* L. Extract Prepared Using an Emulsion–Diffusion Process.” Flavour and Fragrance Journal 28, no. 5: 309–315.

[fsn370486-bib-0036] Farahbakhsh, S. , S. Arbabian , F. Emami , et al. 2011. “Inhibition of Cyclooxygenase Type 1 and 2 Enzyme by Aqueous Extract of *Elaeagnus angustifolia* in Mice.” Basic and Clinical Neuroscience 2, no. 2: 31–37.

[fsn370486-bib-0037] Faramarz, S. , G. Dehghan , and A. Jahanban‐Esfahlan . 2015. “Antioxidants in Different Parts of Oleaster as a Function of Genotype.” BioImpacts: BI 5, no. 2: 79. 26191501 10.15171/bi.2015.09 PMC4492188

[fsn370486-bib-0038] Farzaei, M. H. , R. Bahramsoltani , Z. Abbasabadi , and R. Rahimi . 2015. “A Comprehensive Review on Phytochemical and Pharmacological Aspects of *Elaeagnus angustifolia* L.” Journal of Pharmacy and Pharmacology 67, no. 11: 1467–1480. 10.1111/jphp.12442. 26076872

[fsn370486-bib-0039] Farzaei, M. H. , A. K. Singh , R. Kumar , et al. 2019. “Targeting Inflammation by Flavonoids: Novel Therapeutic Strategy for Metabolic Disorders.” International Journal of Molecular Sciences 20, no. 19: 4957. 10.3390/ijms20194957. 31597283 PMC6801776

[fsn370486-bib-0040] Follstad Shah, J. J. , M. J. Harner , and T. M. Tibbets . 2009. “ *Elaeagnus angustifolia* Elevates Soil Inorganic Nitrogen Pools in Riparian Ecosystems.” Ecosystems 13, no. 1: 46–61. 10.1007/s10021-009-9299-4.

[fsn370486-bib-0041] Fonia, A. , I. R. White , and J. M. White . 2009. “Allergic Contact Dermatitis to Elaeagnus Plant (Oleaster).” Contact Dermatitis 60, no. 3: 178–179. 10.1111/j.1600-0536.2008.01485.x. 19260921

[fsn370486-bib-0042] Fouzat, A. 2021. *Elaeagnus angustifolia* Extract Inhabits Cell Invasion of Human Colorectal Cancer Cells and Increases the Survival Rate of the Drosophila Colon Cancer Model. Qatar University Press.

[fsn370486-bib-0043] Gaskin, J. F. , J. A. Andrés , S. M. Bogdanowicz , et al. 2019. “Russian‐Olive ( *Elaeagnus angustifolia* ) Genetic Diversity in the Western United States and Implications for Biological Control.” Invasive Plant Science and Management 12, no. 2: 89–96.

[fsn370486-bib-0044] Ge, Y. , J. Liu , and D. Su . 2009. “In Vivo Evaluation of the Anti‐Asthmatic, Antitussive and Expectorant Activities of Extract and Fractions From *Elaeagnus pungens* Leaf.” Journal of Ethnopharmacology 126, no. 3: 538–542. 10.1016/j.jep.2009.08.042. 19735714

[fsn370486-bib-0045] Hamidpour, R. , S. Hamidpour , and P. Doostmohamadi . 2019. “Chemistry, Pharmacology and Medicinal Property of Russian Olive ( *Elaeagnus angustifolia* L.).” Cancer Science & Research 6: 1–7.

[fsn370486-bib-0046] Hamidpour, R. , S. Hamidpour , M. Hamidpour , et al. 2017. “Russian Olive ( *Elaeagnus angustifolia* L.): From a Variety of Traditional Medicinal Applications to Its Novel Roles as Active Antioxidant, Anti‐Inflammatory, Anti‐Mutagenic and Analgesic Agent.” Journal of Traditional and Complementary Medicine 7, no. 1: 24–29. 28053884 10.1016/j.jtcme.2015.09.004 PMC5198788

[fsn370486-bib-0047] Heydari Nasrabadi, M. , M. Parsivand , N. Mohammadi , and N. Asghari Moghaddam . 2022. “Comparison of *Elaeagnus angustifolia* L. Extract and Quercetin on Mouse Model of Knee Osteoarthritis.” Journal of Ayurveda and Integrative Medicine 13, no. 2: 100529. 10.1016/j.jaim.2021.10.001. 34862093 PMC8728052

[fsn370486-bib-0048] Hosseinzadeh, H. , M. Ramezani , and N. Namjo . 2003. “Muscle Relaxant Activity of *Elaeagnus angustifolia* L. Fruit Seeds in Mice.” Journal of Ethnopharmacology 84, no. 2–3: 275–278. 10.1016/s0378-8741(02)00331-8. 12648826

[fsn370486-bib-0049] Incedayi, B. , and N. T. Erol . 2023. “Assessment of the Bioaccessibility of *Elaeagnus angustifolia* L. Flour and Its Use in Cracker Formulation.” Plant Foods for Human Nutrition 78, no. 1: 201–206. 10.1007/s11130-022-01041-7. 36622535

[fsn370486-bib-0050] Incilay, G. 2014. “Volatile Composition, Antimicrobial and Antioxidant Properties of Different Parts From *Elaeagnus angustifolia* L.” Journal of Essential Oil Bearing Plants 17, no. 6: 1187–1202.

[fsn370486-bib-0051] Jabeen, A. , A. Sharma , I. Gupta , et al. 2020. “ *Elaeagnus angustifolia* Plant Extract Inhibits Epithelial‐Mesenchymal Transition and Induces Apoptosis via HER2 Inactivation and JNK Pathway in HER2‐Positive Breast Cancer Cells.” Molecules 25, no. 18: 4240. 10.3390/molecules25184240. 32947764 PMC7570883

[fsn370486-bib-0052] Jalalvand, F. , A. Rezaei , B. Badehnoosh , et al. 2021. “The Effects of *Elaeagnus angustifolia* L. on the Thyroid‐Stimulating Hormone, Dehydroepiandrosterone‐Sulfate, Prolactin and Cortisol Levels in Post‐Menopausal Women: A Double‐Blind, Randomized, and Placebo‐Controlled Study.” Frontiers in Pharmacology 12: 654459. 10.3389/fphar.2021.654459. 34305584 PMC8293672

[fsn370486-bib-0053] Karimifar, M. , R. Soltani , V. Hajhashemi , and S. Sarrafchi . 2017. “Evaluation of the Effect of *Elaeagnus angustifolia* Alone and Combined With Boswellia Thurifera Compared With Ibuprofen in Patients With Knee Osteoarthritis: A Randomized Double‐Blind Controlled Clinical Trial.” Clinical Rheumatology 36, no. 8: 1849–1853. 10.1007/s10067-017-3603-z. 28349271

[fsn370486-bib-0054] Katz, G. L. , and P. B. Shafroth . 2003. “Biology, Ecology and Management of *Elaeagnus angustifolia* L.(Russian Olive) in Western North America.” Wetlands 23, no. 4: 763–777.

[fsn370486-bib-0055] Khamzina, A. , J. P. Lamers , and P. L. Vlek . 2009. “Nitrogen Fixation by *Elaeagnus angustifolia* in the Reclamation of Degraded Croplands of Central Asia.” Tree Physiology 29, no. 6: 799–808. 19324691 10.1093/treephys/tpp017

[fsn370486-bib-0056] Khan, S. U. , A. U. Khan , A. U. Shah , et al. 2016. “Heavy Metals Content, Phytochemical Composition, Antimicrobial and Insecticidal Evaluation of *Elaeagnus angustifolia* .” Toxicology and Industrial Health 32, no. 1: 154–161. 10.1177/0748233713498459. 24081630

[fsn370486-bib-0057] Kim, C.‐H. 2021. “Anti–SARS‐CoV‐2 Natural Products as Potentially Therapeutic Agents.” Frontiers in Pharmacology 12: 590509. 34122058 10.3389/fphar.2021.590509 PMC8194829

[fsn370486-bib-0058] Kiseleva, T. , and L. Chindyaeva . 2011. “Biology of Oleaster ( *Elaeagnus angustifolia* L.) at the Northeastern Limit of Its Range.” Contemporary Problems of Ecology 4: 218–222.

[fsn370486-bib-0059] Klich, M. G. 2000. “Leaf Variations in *Elaeagnus angustifolia* Related to Environmental Heterogeneity.” Environmental and Experimental Botany 44, no. 3: 171–183. 10.1016/s0098-8472(00)00056-3. 11064038

[fsn370486-bib-0060] Mamashli, M. , S. Nasseri , Y. Mohammadi , S. Ayati , and A. Zarban . 2022. “Anti‐Inflammatory Effects of N‐Acetylcysteine and *Elaeagnus angustifolia* Extract on Acute Lung Injury Induced by Lambda‐Carrageenan in Rat.” Inflammopharmacology 30, no. 5: 1759–1768. 10.1007/s10787-022-01003-0. 35723848 PMC9207887

[fsn370486-bib-0061] Mazraedoost, S. , G. Behbudi , S. M. Mousavi , and S. A. Hashemi . 2021. “Covid‐19 Treatment by Plant Compounds.” Advances in Applied NanoBio‐Technologies 2, no. 1: 23–33.

[fsn370486-bib-0062] Mohammadhosseini, M. , C. Frezza , A. Venditti , and A. Akbarzadeh . 2019. “Ethnobotany and Phytochemistry of the Genus Eremostachys Bunge.” Current Organic Chemistry 23, no. 17: 1828–1842.

[fsn370486-bib-0063] Mohammadhosseini, M. , C. Frezza , A. Venditti , and S. D. Sarker . 2021. “A Systematic Review on Phytochemistry, Ethnobotany and Biological Activities of the Genus Bunium L.” Chemistry & Biodiversity 18, no. 11: e2100317. 10.1002/cbdv.202100317. 34554642

[fsn370486-bib-0064] Motevalian, M. , M. Shiri , S. Shiri , Z. Shiri , and H. Shiri . 2017. “Anti‐Inflammatory Activity of *Elaeagnus angustifolia* Fruit Extract on Rat Paw Edema.” Journal of Basic and Clinical Physiology and Pharmacology 28, no. 4: 377–381. 10.1515/jbcpp-2015-0154. 28358712

[fsn370486-bib-0065] Natanzi, M. M. , P. Pasalar , M. Kamalinejad , et al. 2012. “Effect of Aqueous Extract of *Elaeagnus angustifolia* Fruit on Experimental Cutaneous Wound Healing in Rats.” Acta Medica Iranica 50, no. 9: 589–596. 23165807

[fsn370486-bib-0066] Nazir, N. , M. Zahoor , and M. Nisar . 2020. “A Review on Traditional Uses and Pharmacological Importance of Genus Elaeagnus Species.” Botanical Review 86: 247–280.

[fsn370486-bib-0067] Niknam, F. , A. Azadi , A. Barzegar , P. Faridi , N. Tanideh , and M. M. Zarshenas . 2016. “Phytochemistry and Phytotherapeutic Aspects of *Elaeagnus angustifolia* L.” Current Drug Discovery Technologies 13, no. 4: 199–210. 10.2174/1570163813666160905115325. 27593387

[fsn370486-bib-0068] Nikniaz, Z. , R. Mahdavi , L. Nikniaz , A. Ebrahimi , and A. Ostadrahimi . 2016. “Effects of *Elaeagnus angustifolia* L. on Lipid Profile and Atherogenic Indices in Obese Females: A Randomized Controlled Clinical Trial.” Journal of Dietary Supplements 13, no. 6: 595–606. 10.3109/19390211.2016.1150933. 26930244

[fsn370486-bib-0069] Nikniaz, Z. , A. Ostadrahimi , R. Mahdavi , A. A. Ebrahimi , and L. Nikniaz . 2014. “Effects of *Elaeagnus angustifolia* L. Supplementation on Serum Levels of Inflammatory Cytokines and Matrix Metalloproteinases in Females With Knee Osteoarthritis.” Complementary Therapies in Medicine 22, no. 5: 864–869. 10.1016/j.ctim.2014.07.004. 25440377

[fsn370486-bib-0070] Nile, S. H. , A. Nile , S. Jalde , and G. Kai . 2021. “Recent Advances in Potential Drug Therapies Combating COVID‐19 and Related Coronaviruses – A Perspective.” Food and Chemical Toxicology 154: 112333. 10.1016/j.fct.2021.112333. 34118347 PMC8189744

[fsn370486-bib-0071] Nistratov, A. V. , V. N. Klushin , E. S. Makashova , and L. V. Kim . 2020. “Production and Evaluation of Properties of Waste‐Based Carbon Adsorbent.” Chemical Engineering Research and Design 160: 551–560.

[fsn370486-bib-0072] Okmen, G. , and O. Turkcan . 2014. “A Study on Antimicrobial, Antioxidant and Antimutagenic Activities of *Elaeagnus angustifolia* L. Leaves.” African Journal of Traditional, Complementary, and Alternative Medicines 11, no. 1: 116–120. 10.4314/ajtcam.v11i1.17. PMC3957251 24653563

[fsn370486-bib-0073] Panahi, Y. , G. H. Alishiri , N. Bayat , S. M. Hosseini , and A. Sahebkar . 2016. “Efficacy of *Elaeagnus angustifolia* Extract in the Treatment of Knee Osteoarthritis: A Randomized Controlled Trial.” EXCLI Journal 15: 203–210. 10.17179/excli2015-639. 27330526 PMC4908661

[fsn370486-bib-0074] Pretty Paint‐Small, V. , K. G. Beck , T. J. Stohlgren , C. S. Brown , and K. A. Sherman . 2013. “Linking Culture, Ecology and Policy: The Invasion of Russian‐Olive ( *Elaeagnus angustifolia* L.) on the Crow Indian Reservation, South‐Central Montana, USA.” Dissertation.

[fsn370486-bib-0075] Sahan, Y. , D. Gocmen , A. Cansev , et al. 2015. “Chemical and Techno‐Functional Properties of Flours From Peeled and Unpeeled Oleaster ( *Elaeagnus angustifolia* L.).” Journal of Applied Botany and Food Quality 88: 34–41.

[fsn370486-bib-0076] Saleh, A. I. , I. Mohamed , A. A. Mohamed , et al. 2018. “ *Elaeagnus angustifolia* Plant Extract Inhibits Angiogenesis and Downgrades Cell Invasion of Human Oral Cancer Cells via Erk1/Erk2 Inactivation.” Nutrition and Cancer 70, no. 2: 297–305. 10.1080/01635581.2018.1412472. 29300111

[fsn370486-bib-0077] Sardar, K. , S. Ali , S. Hameed , et al. 2013. “Heavy Metals Contamination and What Are the Impacts on Living Organisms.” Greener Journal of Environmental Management Public Safety 2, no. 4: 172–179.

[fsn370486-bib-0078] Sarvarian, M. , A. Jafarpour , C. G. Awuchi , A. O. Adeleye , and C. O. R. Okpala . 2022. “Changes in Physicochemical, Free Radical Activity, Total Phenolic and Sensory Properties of Orange ( *Citrus sinensis* L.) Juice Fortified With Different Oleaster ( *Elaeagnus angustifolia* L.) Extracts.” Molecules 27, no. 5: 1530. 10.3390/molecules27051530. 35268631 PMC8912112

[fsn370486-bib-0079] Shabani, M. , A. Rezaei , B. Badehnoosh , et al. 2021. “The Effects of *Elaeagnus angustifolia* L. on Lipid and Glycaemic Profiles and Cardiovascular Function in Menopausal Women: A Double‐Blind, Randomized, Placebo‐Controlled Study.” International Journal of Clinical Practice 75, no. 4: e13812. 10.1111/ijcp.13812. 33145864

[fsn370486-bib-0080] Sharifian‐Nejad, M. S. , and H. Shekarchizadeh . 2019. “Physicochemical and Functional Properties of Oleaster ( *Elaeagnus angustifolia* L.) Polysaccharides Extracted Under Optimal Conditions.” International Journal of Biological Macromolecules 124: 946–954. 10.1016/j.ijbiomac.2018.12.049. 30521891

[fsn370486-bib-0081] Si, C. L. , P. P. Qin , Y. Y. Lu , et al. 2011. “GC‐MS Analysis of Chemical Composition and Free Radical Scavenging Activity of *Elaeagnus angustifolia* Bark.” Advanced Materials Research 183: 854–858.

[fsn370486-bib-0082] Sohail, M. I. , A. Siddiqui , N. Erum , and M. Kamran . 2021. “Phytomedicine and the COVID‐19 Pandemic.” In Phytomedicine, 693–708. Elsevier.

[fsn370486-bib-0083] Srivastava, A. K. , A. Kumar , and N. Misra . 2020. “On the Inhibition of COVID‐19 Protease by Indian Herbal Plants: An In Silico Investigation.” arXiv preprint arXiv:.03411.

[fsn370486-bib-0084] Stubbendieck, J. L. , M. J. Coffin , and L. M. Landholt . 2003. Weeds of the Great Plains. Nebraska Department of Agriculture, Bureau of Plant Industry.

[fsn370486-bib-0085] Sytar, O. , M. Brestic , S. Hajihashemi , et al. 2021. “COVID‐19 Prophylaxis Efforts Based on Natural Antiviral Plant Extracts and Their Compounds.” Molecules 26, no. 3: 727. 33573318 10.3390/molecules26030727 PMC7866841

[fsn370486-bib-0086] Tang, Y. , X. He , G. Liu , et al. 2023. “Effects of Different Extraction Methods on the Structural, Antioxidant and Hypoglycemic Properties of Red Pitaya Stem Polysaccharide.” Food Chemistry 405, no. Pt A: 134804. 10.1016/j.foodchem.2022.134804. 36356363

[fsn370486-bib-0087] Tehranizadeh, Z. A. , A. Baratian , and H. Hosseinzadeh . 2016. “Russian Olive ( *Elaeagnus angustifolia* ) as a Herbal Healer.” BioImpacts: BI 6, no. 3: 155. 27853679 10.15171/bi.2016.22 PMC5108988

[fsn370486-bib-0088] Tolkachev, O. N. , E. A. Abizov , E. V. Abizova , and S. D. Mal'Tsev . 2008. “Phytochemical Study of the Bark of Some Plants of the Elaeagnaceae Family as a Natural Source of β‐Carboline Indole Alkaloids.” Pharmaceutical Chemistry Journal 42, no. 11: 630–632.

[fsn370486-bib-0089] Torbati, M. , S. Asnaashari , and F. Heshmati Afshar . 2016. “Essential Oil From Flowers and Leaves of *Elaeagnus angustifolia* (Elaeagnaceae): Composition, Radical Scavenging and General Toxicity Activities.” Advanced Pharmaceutical Bulletin 6, no. 2: 163–169. 10.15171/apb.2016.023. 27478777 PMC4961973

[fsn370486-bib-0090] Vahidi‐eyrisofla, N. , S. J. Rastin , F. Taghvaei , M. Ahmadifar , and A. M. Eini . 2015. “Effect of *Peganum harmala* L. on Lipid Metabolism and Changes Cyp7a1 Gene Expression in Male Wistar Rat.” Journal of Renewable Natural Resources Bhutan 1608: 4330.

[fsn370486-bib-0091] Wang, Y. A. , T. Guo , C.‐M. Zhao , J.‐Y. Li , P. Zhao , and M.‐T. Fan . 2014. “Changes in Total Phenolic and Flavonoid Contents and Antioxidant Activities of the Fruit From *Elaeagnus angustifolia* During an 80‐Day Study Period.” Agro Food Industry Hi‐Tech 25: 7–10.

[fsn370486-bib-0092] Wei, C. , L. Yao , Y. Zhang , et al. 2023. “Structural Characterization of Peach Gum Polysaccharide and Its Effects on the Regulation of DSS‐Induced Acute Colitis.” International Journal of Biological Macromolecules 225: 1224–1234. 10.1016/j.ijbiomac.2022.11.183. 36427612

[fsn370486-bib-0093] Wink, M. 2020. “Potential of DNA Intercalating Alkaloids and Other Plant Secondary Metabolites Against SARS‐CoV‐2 Causing COVID‐19.” Diversity 12, no. 5: 175.

[fsn370486-bib-0094] Yalcin, G. , and O. Sogut . 2014. “Influence of Extraction Solvent on Antioxidant Capacity Value of Oleaster Measured by ORAC Method.” Natural Product Research 28, no. 18: 1513–1517. 10.1080/14786419.2014.913243. 24783990

[fsn370486-bib-0095] Yuca, H. , H. Ozbek , L. O. Demirezer , H. G. Kasil , and Z. Guvenalp . 2021. “Trans‐Tiliroside: A Potent Alpha‐Glucosidase Inhibitor From the Leaves of *Elaeagnus angustifolia* L.” Phytochemistry 188: 112795. 10.1016/j.phytochem.2021.112795. 34044297

[fsn370486-bib-0096] Zakaria, Z. Z. , Y. Ahen , N. H. Ahmed , S. Sokary , H. Bawadi , and M. Al‐Asmakh . 2023. “Exploring *Elaeagnus angustifolia* : A Comprehensive Review of Its Potential in Cancer Treatment and Management.” Preprints.

[fsn370486-bib-0097] Zeinalzadeh, S. , A. A. Mohagheghzadeh , F. Ahmadinezhad , and M. Akbarzadeh . 2019. “Comparison of the Effect of *Elaeagnus angustifolia* Flower Capsule and Sildenafil Citrate Tablet Female Sexual Interest/Arousal Disorder in Clinical Trial Study.” Journal of Family Medicine and Primary Care 8, no. 11: 3614–3620. 10.4103/jfmpc.jfmpc_525_19. PMC6881950 31803662

[fsn370486-bib-0098] Zeka, K. , K. Ruparelia , R. R. J. Arroo , R. Budriesi , and M. Micucci . 2017. “Flavonoids and Their Metabolites: Prevention in Cardiovascular Diseases and Diabetes.” Diseases 5, no. 3: 19. 32962323 10.3390/diseases5030019 PMC5622335

[fsn370486-bib-0099] Zhang, W. , Y. Guo , Y. Cheng , W. Yao , and H. Qian . 2023. “Neuroprotective Effects of Polysaccharide From Sparassis Crispa on Alzheimer's Disease‐Like Mice: Involvement of Microbiota‐Gut‐Brain Axis.” International Journal of Biological Macromolecules 225: 974–986. 10.1016/j.ijbiomac.2022.11.160. 36402384

[fsn370486-bib-0100] Zhao, L. Y. , Q. J. Lan , Z. C. Huang , L. J. Ouyang , and F. H. Zeng . 2011. “Antidiabetic Effect of a Newly Identified Component of *Opuntia dillenii* Polysaccharides.” Phytomedicine 18, no. 8–9: 661–668. 10.1016/j.phymed.2011.01.001. 21300531

[fsn370486-bib-0101] Zhou, X. , J. R. Brandle , M. M. Schoeneberger , and T. Awada . 2007. “Developing Above‐Ground Woody Biomass Equations for Open‐Grown, Multiple‐Stemmed Tree Species: Shelterbelt‐Grown Russian‐Olive.” Ecological Modelling 202, no. 3–4: 311–323.

